# The Corset Splint

**Published:** 1887-01

**Authors:** John Thad. Johnson

**Affiliations:** Late Professor of Surgery, Southern Medical College, Atlanta, Ga.


### THE CORSET SPLINT.

### A READY AND EFFICIENT APPARATUS FOR FRACTURES.

BY JNO. THAD. JOHNSON, M. D., LATE PROFESSOR OF SURGERY, SOUTHERN MEDICAL COLLEGE, ATLANTA, GA.

It may seem that there is already an over-supply of material for splints; yet, with this multitude of material mentioned in text-books, the practitioner is often at a loss for something that may be easily procured. One does not wish to rely upon the fanciful appliances of the instrument-maker. Wooden splints, as made impromptu, are bungling, and worse things may be said of them—starch, dextrine, etc., have fallen into "innocuous desultude." The disadvantages of plaster of Paris, in many instances, are apparent.

Wherever the patient may be, it is easy to obtain a few slender twiggs, or reeds, or whalebone strips, or "white-oak splits," such as are used in many sections for basket-making.

It is an easy matter to run a number of seams through a piece of muslin, once folded; this to be of length and width to correspond with the injured limb. These seams must be from one-eighth to one inch apart, as necessary, thus making as many pockets or compartments of desired length and width as the limb may demand.

Through each of these pockets or sections we run a twig or splint. The twig may be willow, peach, apple, anything slender and pliable, and it should be stripped of bark.

Having thus constructed our corset material, it is easy to make of it an accurately fitting apparatus by first taking a pattern of the limb in thick paper, usually in two lateral sections. This pattern laid upon the material enables us to cut a closely-fitting jacket or corset.

These lateral halves may be stitched or hinged together so the jacket may be easily removed and applied.

A layer of cotton wadding, cloth or other suitable material must be placed between skin and splint. This need not be thick, but we must be careful of the edges of the apparatus.

In certain regions, near the wrist-joint, as an instance, a thicker padding may be placed, for when the bone-fragments are properly in position, this cushion, firmly pressed by splint and bandage, moulds itself to the inequalities of surface much better than the fancily-carved and curved instruments offered us by excessively ingenious surgeons and instrument-makers.

A roller bandage, bandage of Scultetus, separate bandage strips, or rubber bands, serve to retain the apparatus in place. To make the corset fit more perfectly, we might attach hooks-and-eyes, or lacing eyelets.

This corset-splint is not proposed as a substitute for every other apparatus; some requirements it would fail to meet. But in most fractures of the extremities, it will serve us either as a temporary device or permanent dressing. Among other advantages the facility with which it can be prepared and the general presence of its *ingredients* would especially recommend it to the attention of those who must provide apparatus impromptu from meagre resources.

A sheet of this material might be kept on hand for emergencies, and easily fitted to the case in hand. However, as it is so readily prepared, and as it might lose some of its pliability by age, we would generally prefer to make it when there is occasion for its use.

Cases are met with where we hesitate to apply plaster of Paris at once; the corset-splint will serve us well until there is no longer fear of danger. Further, it will often do away with the necessity for the plaster application. In fact, there are many instances where it proves an excellent temporary support, even if something else is to be applied at a later period.

This material will afford a good substitute for the four short splints often used on the thigh, when a fracture of the femur is treated by weight and pulley.

It makes a good and ready support for hand and arm where the upper limb must be suspended in a sling. Liquid applications, hot or cold, may be made through it, an advantage not possessed by otherwise excellent apparatuses.

Instead of wood or whalebone, strips of tin, or zinc, or leather, or wire of different sizes, may meet special indications.

As constructed of the metal strips, it would give strength to plaster bandages and diminish bulk and weight. In such case, crinoline might answer a better purpose than thicker muslin. Made of this material it answers well for hand or fingers. Constructed of leather strips, it gives support to certain injured joints, such as a sprained ankle, for instance. Indeed, when made of any of the last proposed strips, it answers fairly well even for elbow and shoulder. When it is desired to give increased strength, two or more layers made of very slender twigs may serve a better purpose than one thickness of larger growth. As announced in the outset, this is proposed, not as wonderful or magical in effects, but as giving efficient service where other splints cannot be procured.

It has been suggested to me that the idea or principle of this corset-splint is similar to the strips of wood glued on kid or other skin, but there is nothing in common between the two. One is stiff, unyielding, and must be prepared by special skill, and is not always at hand. Liquid applications would injure it. The other is cheap, easily obtained, quickly prepared, accurate in fit, not injured by liquids, with other advantages unnecessary to mention.

